# Risk Factors of Low APGAR Score in Japanese Full-term Deliveries: A Case-control Study

**DOI:** 10.2188/jea.12.320

**Published:** 2007-11-30

**Authors:** Machi Suka, Hiroki Sugimori, Makoto Nakamura, Kazumoto Haginiwa, Katsumi Yoshida

**Affiliations:** 1 Department of Preventive Medicine, St. Marianna University School of Medicine; 2 Department of Obstetrics, St. Marianna University School of Medicine

**Keywords:** case-control studies, APGAR score, risk factors, induced abortion, preeclampsia

## Abstract

To elucidate maternal characteristics and pregnancy complications associated with low APGAR score, a case-control study of low APGAR score was conducted under matching both gestational age and route of delivery, in full-term deliveries at a Japanese hospital with 102 cases and 204 controls. Previous induced abortion and occurrence of preeclampsia were more frequently observed in the low APGAR score cases. In the multiple conditional logistic regression analysis, each of these factors more than doubled the risk of low APGAR score. Even if only those without perinatal troubles were included in the analysis, previous induced abortion was recognized as an independent risk factor of low APGAR score (odds ratio=2.68, 95% confidence interval:1.01-7.04). Despite of the potential limitations of this study, previous induced abortion might be a useful predictor of adverse state of newborn infant.

Japanese Ministry of Health and Welfare has implemented antenatal care services under the Maternal and Child Health Law, which consists of regular health checkups for pregnant women and issue of maternity passbook. If it is possible to predict adverse pregnancy outcome during the antenatal care, obstetricians will be able to take appropriate countermeasures for high-risk pregnant women; the early preventative actions may lead to maternal and child well-being.

APGAR score is a reliable indicator for evaluating the state of newborn infant.^1^^,^^2^ Each of the five components, heart rate, respiratory effort, muscle tone, reflex irritability, and color, is assessed and assigned a value of 0 to 2. The APGAR score is the sum of the five components, and below 8 of this score suggests adverse pregnancy outcome.^2^^,^^3^ Knowledge about risk factors of low APGAR score may contribute to improving the antenatal care based on better identification of high-risk pregnant women. This study aimed to elucidate maternal characteristics and pregnancy complications associated with low APGAR score in Japanese full-term deliveries.

## SUBJECTS AND METHODS

A case-control study of low APGAR score was conducted in all full-term deliveries between January 1, 1993 and December 31, 1995 at a Japanese hospital, which has played a central role in community health of Kawasaki area. We reviewed three-year delivery records and abstracted information on maternal characteristics, pregnancy complications and pregnancy outcome, which were entered into a computerized data base. We obtained eligible 2,425 full-term deliveries whose gestational age, route of delivery and a 1-minute APGAR score were completed. Among them, there were 102 cases with below 8 of a 1-minute APGAR score (low APGAR score).^2^^,^^3^ For each of the low APGAR score cases, two controls were randomly selected with matching both gestational age (weeks) and route of delivery (vaginal delivery or cesarean delivery) from the rest of the full-term deliveries. Table 1 shows the distribution of APGAR score.

**Table 1.** Distribution of APGAR score.

	APGAR score											total

	0	1	2	3	4	5	6	7	8	9	10	
cases	5	3	5	5	2	13	14	55	0	0	0	102
controls	0	0	0	0	0	0	0	0	28	155	21	204

Information on maternal characteristics had been collected through both the questionnaire and the obstetricians’ interview at the first antenatal visit. Occurrence of pregnancy complications had been recognized according to the prescribed hospital guidelines. The factors analyzed in this study were: low maternal age (≤ 18 years old), high maternal age (≥ 35 years old), short statue (< 150 cm), progravid thin (BMI < 18 kg/m^2^, the 10th percentile for all deliveries), progravid obesity (BMI > 26kg/m^2^, the 90th percentile for all deliveries), smoking (any), drinking (any), marital status (unmarried), previous pregnancy (≥ 1), previous delivery (≥ 1), previous spontaneous abortion (≥ 1), previous induced abortion (≥ 1), occurrence of threatened abortion, occurrence of anemia, occurrence of preeclampsia, and medical complications (hypertension, diabetes, asthma, cardiac disease, and renal disease). These factors are usually obtained from antenatal care in the first and second trimester pregnancy.

Statistical analyses were performed with the Statistical Analysis Systems (SAS, version 6.12). Proportions of the factors in the cases and the controls were compared by McNemar’s test. When any values in the cases and the controls were zero, 0.5 was added to each value for calculating odds ratio. Adjusted odds ratios (ORs) and their corresponding 95% confidence intervals (CIs) were calculated using conditional logistic regression models,^4^ which incorporated the factors with statistically significant differences between the cases and the controls (p<0.05 with McNemar’s test).

## RESULTS

Table 2 shows the comparisons of maternal characteristics and pregnancy complications between the low APGAR score cases and the controls. Previous induced abortion and occurrence of preeclampsia were more frequently observed in the low APGAR score cases. There were no significant differences in factors regarding maternal age, physical constitutions, behavioral habits, and medical complications.

**Table 2.** Comparisons of maternal characteristics and pregnancy complications between the low APGAR score cases and the controls (by McNemar’s test).

variable	proportion (%)		odds ratio	(95% CI)

	cases (n=102)	controls (n=204)		
low maternal age ( ≤ 18 y.o.) † ¶	0	1	0.72	(0.03-17.95)
high maternal age ( ≥ 35 y.o.) †	23	15	1.62	(0.89-2.95)
short statue (< 150 cm)	2	6	0.29	(0.07-1.22)
progravid thin (BMI < 18 kg/m^2^) ‡	15	17	0.91	(0.47-1.78)
progravid obese (BMI > 26 kg/m^2^) ‡	16	9	1.89	(0.91-3.92)
smoking (any)	21	15	1.54	(0.83-2.86)
drinking (any)	39	45	0.80	(0.49-1.30)
marital status (unmarried) ¶	1	0	6.24	(0.25-154.73)
previous pregnancy ( ≥ 1)	54	54	1.00	(0.62-1.61)
previous delivery ( ≥ 1)	35	41	0.78	(0.48-1.28)
previous spontaneous abortion ( ≥ 1)	11	13	0.79	(0.38-1.67)
previous induced abortion ( ≥ 1)	24	14	1.86	(1.02-3.38)
occurrence of threatened abortion	6	8	0.73	(0.28-1.93)
occurrence of anemia	32	39	0.74	(0.45-1.22)
occurrence of preeclampsia	28	16	2.03	(1.15-3.60)

† : refered to those with a maternal age of 19-34 y.o..

‡ : refered to those with a BMI of 18-26 kg/m^2^.

¶ : 0.5 was added to each value for calculating odds ratios.

Table3 shows the results of multiple conditional logistic regression analyses. Previous induced abortion and occurrence of preeclampsia more than doubled the risk of low APGAR score. According to our preliminary results, APGAR score could be directly affected by the occurrence of perinatal troubles such as abruptio placentae and fetal distress. Even if only 46 pairs (46 cases and 92 controls) without these perinatal troubles were included in the analysis, previous induced abortion was recognized as an independent risk factor of low APGAR score (OR=2.68, 95% CI:1.01-7.04).

**Table 3.** Adjusted odds ratios (95% confidence intervals) for risk of low APGAR score. ^¶^

variable	total	only without perinatal troubles
	(102 pairs)	(46 pairs)
previous induced abortion ( ≥ 1)	2.10	2.68
	(1.11-4.00)	(1.01-7.14)
		
occurrence of preeclampsia	2.18	1.81
	(1.21-3.95)	(0.77-4.23)

¶ calculated using conditional logistic regression models, incorporated the factors listed in the table.

## DISCUSSION

APGAR score has been used to evaluate the state of newborn infant throughout the world for almost 50 years. Although some limitations of the scoring system have been pointed out,^5^^,^^6^ it should be noted that, at present, there is no single measure that immediately represents fetal or neonatal condition but APGAR score.^1^ Low APGAR score indicates the adverse state of newborn infant, in which obstetricians must take all possible measures to ensure resuscitation.^1^^,^^2^^,^^5^ Risk factors of low APGAR score may be useful information to reduce the risk of adverse pregnancy outcome.

Among the 12 maternal characteristics and 3 pregnancy complications analyzed in this study, previous induced abortion and occurrence of preeclampsia more than doubled the risk of low APGAR score. This result suggests that these factors should be considered to improve risk assessment and risk management during antenatal care. It is a well-known fact that preeclampsia may lead to further pregnancy complications (e.g. eclampsia, abruptio placentae, growth retardation, fetal distress, stillbirth), and screening for preeclampsia at the first antenatal visit is urged by U.S. Preventive Services Task Force.^7^ Little is known about the effect of previous induced abortion on APGAR score, but we found that previous induced abortion was associated with an increased risk of low APGAR score regardless of the occurrence of perinatal troubles. This obstetrical history might be a useful predictor of adverse state of newborn infant.

Some studies showed that the effect of previous induced abortion on pregnancy outcome such as preterm and low birthweight could be affected by number of induced abortions,^8^^-^^10^ method of abortion (curettage or vacuum aspiration),^8^^-^^12^ timing of abortion (completed gestational weeks),^8^^,^^9^ and interpregnancy interval.^8^^,^^9^ It is possible that some of these factors affect the findings of this study. Further studies, taking into account these factors, may be required to have a better understanding about the effect of previous induced abortion on APGAR score.

This study has the following potential limitations. First, we could not consider the difference between planned cesarean and urgent cesarean for matching route of delivery. Most of the urgent cesarean deliveries were caused by perinatal troubles such as abruptio placentae and fetal distress; of the 48 full-term deliveries by urgent cesarean obtained from our data base, more than 70% had experienced these troubles. Urgent cesarean deliveries were more likely to have low APGAR score. However, the sub-analysis in those without perinatal troubles confirmed the association between previous induced abortion and low APGAR score. Second, we could not control for fetal presentation. An early study showed that breech presentation was associated with low APGAR score.^2^ However, perinatal care techniques have improved over time. To prevent troubles in labor, breech presentation often leads to cesarean delivery,^13^ and this study was conducted under matching route of delivery. Finally, we could not control for partuient factors such as duration of labor and forceps or vacuum extraction. These factors might have more direct influences on the state of newborn infant and lower APGAR score.

In summary, previous induced abortion and occurrence of preeclampsia more than doubled the risk of low APGAR score. Moreover, previous induced abortion was associated with an increased risk of low APGAR score regardless of the occurrence of perinatal troubles. Despite of the potential limitations of this study, previous induced abortion might be a useful predictor of adverse state of newborn infant. In this study, we focused on a 1-minute APGAR score, which is known as a useful indicator for assessing the need for resuscitation. Quite a number of cases with low APGAR score at 1 minute have high APGAR score at 5 minutes and later,^3^^,^^14^ and a 5-minute APGAR score is known as a useful indicator of neonatal mortality and morbidity.^3^^,^^15^^-^^17^ Unfortunately, we had no data on a 5-minute APGAR score. In future, we will examine the effects of previous induced abortion and occurrence of preeclampsia on a 5-minute APGAR score.

## References

[1] Papile LA. The Apgar score in the 21st century. N Engl J Med 2001;344:519-20. 11172195 10.1056/NEJM200102153440709

[2] Apgar V, Holaday DA, James LS, Weisbort IM. Evaluation of the newborn infant: second report. JAMA 1958;168:1985-8. 10.1001/jama.1958.03000150027007 13598635

[3] Drage JS, Kennedy C, Schwarz BK. The Apgar score as an index of neonatal mortality: a report from the collaborative study of cerebral palsy. Obstet Gynecol 1964;24:222-30. 14199529

[4] Breslow NE, Day NE. Statistical methods in cancer research Vol.l. The analysis of case-control studies. IARC scientific publications, Lyon 1994. 7216345

[5] American Academy of Pediatrics Committee on Fetus and Newborn: Use and abuse of the Apgar score. Pediatrics 1986;78:1148-9. 3786042

[6] Jepson HA, Talashek ML, Tichy AM. The Apgar score: evolution, limitations, and scoring guidelines. Birth 1991;18:83-92. 1718292 10.1111/j.1523-536x.1991.tb00065.x

[7] U.S. Preventive Services Task Force. Part F. Prenatal disorders. In. U.S. Preventive Services Task Force. Guide to clinical preventive services, second edition. Williams & Wilkins, Baltimore 1996.

[8] Zhou W, Sorensen HT, Olsen J. Induced abortion and subsequent pregnancy duration. Obstet Gynecol 1999;94:948-53. 10576181 10.1016/s0029-7844(99)00440-8

[9] Zhou W, Sorensen HT, Olsen J. Induced abortion and low birthweight in the following pregnancy. Int J Epidemiol 2000;29:100-6. 10750610 10.1093/ije/29.1.100

[10] Henriet L, Kaminski M. Impact of induced abortions on subsequent pregnancy outcome: the 1995 French national perinatal survey. Br J Obstet Gynecol 201;108:1036-42. 10.1111/j.1471-0528.2001.00243.x 11702834

[11] Hogue CJR. Impact of abortion on subsequent fecundity. Clin Obstet Gynaecol 1986;13:95-103. 3519040

[12] Atrash HK, Hogue CJ. The effect of pregnancy termination on future reproduction. Baillieres Clin Obstet Gynaecol 1990;4:391-405. 2225607 10.1016/s0950-3552(05)80234-2

[13] Committee on Obstetric Practice. ACOG committee opinion: mode of term single breech delivery. Obstet Gynecol 2001;98:1189-90. 11755586 10.1016/s0029-7844(01)01708-2

[14] Jonas O, Chan A, Macharper T, Roder D. Pregnancy and perinatal factors associated with persistently low Apgar scores: an analysis of the birth records of infants born in South Australia. Eur J Epidemiol 1990;6:136-41. 2361537 10.1007/BF00145785

[15] Casey BM, McIntire DD, Leveno KJ. The continuing value of the Apgar score for the assessment of newborn infants. N Engl J Med 2001;344:467-71. 11172187 10.1056/NEJM200102153440701

[16] Thorngren-Jerneck K, Herbst A. Low 5-minute Apgar score: a population-based register study of 1 million term births. Obstet Gynecol 2001;98:65-70. 11430958 10.1016/s0029-7844(01)01370-9

[17] Moster D, Lie RT, Irgens LM, Bjerkedal T, Markestad T. The association of Apgar score with subsequent death and cerebral palsy: a population-based study in term infants. J Pediatr 2001;138:798-803. 11391319 10.1067/mpd.2001.114694

